# Panaxatriol saponins promotes angiogenesis and enhances cerebral perfusion after ischemic stroke in rats

**DOI:** 10.1186/s12906-017-1579-5

**Published:** 2017-01-23

**Authors:** Zhen Hui, Du-Juan Sha, Su-Lei Wang, Chao-Sheng Li, Jian Qian, Jing-Qing Wang, Yang Zhao, Jing-Hua Zhang, Hong-Yu Cheng, Hui Yang, Lin-Jie Yu, Yun Xu

**Affiliations:** 1 Department of Neurology, Nanjing Drum Tower Hospital Clinical College of Traditional Chinese and Western Medicine, Nanjing University of Chinese Medicine, Nanjing, 210008 Jiangsu People’s Republic of China; 2 Department of Neurology, Drum Tower Hospital, Medical School of Nanjing University, 321 ZhongShan Road, Nanjing City, Jiangsu Province 210008 People’s Republic of China; 3 Department of Neurology, The Third Affiliated Hospital of Nanjing University of Chinese Medicine, Nanjing, 210001 Jiangsu People’s Republic of China; 4 Department of Emergency, Drum Tower Hospital, Medical School of Nanjing University, Nanjing, 210008 Jiangsu People’s Republic of China; 5 The State Key Laboratory of Pharmaceutical Biotechnology, Nanjing University, Nanjing, 210008 Jiangsu People’s Republic of China; 6 Jiangsu Key Laboratory for Molecular Medicine, Nanjing University Medical School, Nanjing, 210008 Jiangsu People’s Republic of China; 7 Jiangsu Province Stroke Center for Diagnosis and Therapy, Nanjing, 210008 People’s Republic of China; 8 Nanjing Neuropsychiatry Clinic Medical Center, Nanjing, 210008 People’s Republic of China

**Keywords:** Panaxatriol saponins, Ischemic stroke, Angiogenesis, Cerebral perfusion

## Abstract

**Background:**

Panaxatriol saponins (PTS), an extract from the traditional Chinese herb Panax notoginseng, which has been used to treat ischemic stroke for many years in China. However, the mechanism underlying the effects of PTS remains unclear. This study aimed to determine whether PTS can protect against ischemic brain injury by promoting angiogenesis and to explore the possible mechanism by which it promotes angiogenesis.

**Methods:**

Middle cerebral artery occlusion (MCAO) was induced in rats, and neurological deficit scores and brain infarct volumes were assessed. Micro-Positron emission tomography (PET) was adopted to assess cerebral perfusion, and real-time PCR and western blotting were used to evaluate vascular growth factor and Sonic hedgehog (Shh) pathway component levels. Immunofluorescence staining was used to determine capillary densities in ischemic penumbrae.

**Results:**

We showed that PTS improved neurological function and reduced infarct volumes in MCAO rats. Micro-PET indicated that PTS can significantly increase ^18^F-fluorodeoxyglucose (^18^F-PDG) uptake by ischemic brain tissue and enhance cerebral perfusion after MCAO surgery. Moreover, PTS was able to increase capillary densities and enhance angiogenesis in ischemic boundary zones and up-regulate vascular endothelial growth factor (VEGF) and Angiopoietin-1 (Ang-1) expression by activating the Shh signaling pathway.

**Conclusion:**

These findings indicate that PTS exerts protective effects against cerebral ischemic injury by enhancing angiogenesis and improving microperfusion.

**Electronic supplementary material:**

The online version of this article (doi:10.1186/s12906-017-1579-5) contains supplementary material, which is available to authorized users.

## Background

Stroke is the second-most fatal disease worldwide [1], and ischemic stroke is the most common cause of stroke [2]. Restoring cerebral blood flow and saving dying neurons are considered effective therapies for ischemic brain injury. Thrombolytic therapy with recombinant tissue plasminogen activator (rt-PA) is approved by the FDA for treating acute cerebral infarctions [3], but the majority of patients can not benefit from it due to its narrow treatment time window and association with hemorrhagic complications. Therefore, new therapies must be developed to treat the large numbers of patients suffering from ischemic brain injury. Angiogenesis, the formation of new blood vessels from preexisting vessels [4], can improve tissue microperfusion in ischemic boundary regions. A previous study showed that patients with higher penumbra vascular densities can survive for longer periods of time after ischemic stroke [5]. In addition, emerging evidence suggests that angiogenesis can reduce rodent cerebral infarction volumes and improve neurological function [6–8].

Throughout the process of angiogenesis, the VEGF/VEGFR-2 and Ang-1/Tie-2 systems participate in vessel formation and maturation. A previous study showed that the VEGF and Ang-1 systems can be co-regulated by Sonic hedgehog (Shh) signaling in the setting of ischemic injury [9]. Shh is a representative morphogen involved in neural development and vessel formation during embryogenesis [10, 11]. Shh also mediates angiogenesis during postnatal life in the setting of pathological conditions [12–14], a subject that has been widely studied. Shh can induce robust angiogenesis by regulating VEGF and Ang-1 expression in interstitial mesenchymal cells [15], and a recent study reported that Shh can reduce brain infarct volumes and enhance post-ischemic angiogenesis in MCAO models [16]. Moreover, Shh pathway inhibition results in decreased VEGF, Ang-1 and CD31 expression in adult mouse hearts [17].

Panaxatriol saponins (PTS), an extract from the traditional Chinese herb Panax notoginseng, is a monomer compound that is mainly composed of ginsenoside Rg1, notoginsenoside R1, and ginsenoside Re. PTS has been used to treat ischemic stroke for many years in China. However, the mechanism underlying the effects of PTS remains unclear. Clinical research confirmed that PTS combined with low dose of aspirin can significantly ameliorate neurological deficit and activities of daily living in patients with light and moderate ischemic stroke [18]. A previous study showed that PTS protects against the effects of cerebral ischemia by reducing cerebral edema and up-regulating heat shock protein70 (HSP70) expression [19]. A recent study found that PTS can attenuate oxygen-glucose-induced injury in PC12 cells via PI3K/AKT signaling pathway and Nrf2 signaling pathway activation [20]. Meanwhile, PTS provided neuroprotection against dopaminergic neurons loss in 1-methy-4-phenyl-1,2,3,6-tetrahypdropyridine(MPTP) mouse model through increasing thioredoxin-1(Trx-1) expression and inhibiting mitochondria-mediated apoptosis [21]. Furthermore, the main ingredients of PTS, ginsenoside Rg1, notoginsenoside R1, and ginsenoside Re, are believed to exert pro-angiogenic effects in human umbilical vein endothelial cells (HUVECs) and zebrafish chemical-induced blood vessel loss models [22]. In the current study, we investigated whether PTS can promote angiogenesis and the mechanisms underlying its effects in a rodent stroke model.

## Methods

### Animals

All animal protocols were approved by the Experimental Animal Administration Committee of Nanjing University. Amounting to 210 male Sprague-Dawley rats (250–300 g) were provided by the Animal Center of Nanjing Drum Tower Hospital. The rats were individually caged under specific pathogen-free (SPF) conditions and 12-h light-dark cycles. They were allowed free access to water and standard rodent food. All operations were performed under anesthesia induced by 10% chloral hydrate (350 mg/kg) to minimize suffering during the study.

### Transient middle cerebral artery occlusion (tMCAO) in rats and drug treatment

The MCAO model was induced as described in a previous study [23]. Focal ischemic infarction was produced in the right middle cerebral artery territory. Briefly, a 4-0 surgical nylon suture with a heat-rounded tip was inserted into the lumen of the internal carotid artery from the external carotid artery to block the origin of the right MCA (middle cerebral artery). After 2 h of ischemia, the filament was withdrawn to allow reperfusion. Heart rate and blood pressure were monitored during surgery. Sham-operated rats were subjected to the same procedure without middle cerebral artery occlusion. PTS was obtained from Huasun Group Co., LTD (Sichuan, China). The ginsenoside Rg1, notoginsenoside R1, and ginsenoside Re are the main components of PTS with the concentrations of 50, 11 and 6% respectively, as shown in Additional file 1: Figure S1. In a previous study, different doses of PTS (25, 50 and 100 mg/kg/day) were used, triphenyltetrazolium chloride (TTC) staining and Longa scores showed that the dose of 50 mg/kg/day significantly reduced rats infarct volumes and improved neurological deficit scores, but there is no different between 100 mg/kg/day and 50 mg/kg/day, as shown in Additional file 2: Figure S2. Thus, the PTS diluted with saline water into 0.5%, and 50 mg/kg/day PTS was used in this study.

The rats were randomized divided into the following three groups: (1) the Sham group: sham-operated rats i.p. injected with an equal volume of sterile saline; (2) the vehicle group: MCAO rats i.p. injected with an equal volume of sterile saline after sugery and once daily; and (3) the PTS treatment group: MCAO rats i.p. injected with PTS after sugery and once daily, the drug or saline first administrated time was at 2 h latter after sugery. Rats in each group were sacrificed at 1, 3, 7, and 14 days after surgery for subsequent studies. To label proliferating cells, we intraperitoneally injected bromodeoxyuridine (Brdu, 50 mg/kg, sigma) within the first 24 h after surgery and once daily thereafter for 14 days. To explore the mechanism underlying PTS-induced angiogenesis, cyclopamine, a specific Shh pathway inhibitor, was dissolved in 2-hydroxypropyl-β-cyclodextrin (HPβCD) solution (1 mg/ml cyclopamine dissolved in 45% HPβCD). The rats were treated with cyclopamine (10 mg/kg) [24] via i.p. injections within the first 24 h after surgery and once daily for 7 consecutive days.

### Neurological functional assessments

Neurological functional evaluations were performed at 6 h, 1, 3, 7 and 14 days after reperfusion, and the evaluators were blinded to the experimental conditions. Neurological function was assessed using the following modified Longa five-point scale scoring system: 0: no neurological deficits; 1: left forelimb flexion; 2: spontaneous left circling; 3: falls to the left; and 4: no spontaneous movement or loss of consciousness. Rats with scores of 1–3 points were used in this study.

### Measurement of brain infarct volumes

At 1, 3 and 7 days after surgery, the rats were sacrificed under anesthesia. Their brains were sliced into 2.0 mm sections and immersed in 2% TTC (Sigma, USA) for 30 min and then fixed in 4% paraformaldehyde overnight at 4 °C. Normal brain tissue appeared red, and infarcted tissue appeared in pale gray. The slices were photographed, and infarct volumes were calculated using Image-Pro Plus 6.0 software (Media Cybernetics) by a blinded investigator. To determine if cerebral edema was present, the infarct volumes were presented as percentages of the volumes of the contralateral hemispheres. At 14 days after surgery, the rats were anesthetized and perfused with 0.9% saline, and their brains were fixed in 4% paraformaldehyde before being cut into 30 μm coronal sections. One of every 7 consecutive slices was randomly selected for neuronal staining. Infarcted regions were defined as those with absent neuronal staining.

### Micro-PET

Micro-PET information was recorded after cerebral reperfusion at 1 and 14 days after surgery by a blinded investigator. Before PET, the rats were deprived of food for 12 h to enhance brain FDG uptake. FDG (1.0–1.5 mCi/kg) was injected via the tail vein under anesthesia. The injection time, FDG dose, residual dose measurement, and measuring residual time were recorded. FDG was allowed to circulate in the blood for 60 min, and then the animals were fixed on the special scanning bed for a 10 min micro-PET scan. Images obtained using a small-animal PET scanner (Inveon, Germany SIMENS company), and the OSEM 3D format was used to acquire regions of interest.

### Real-time PCR

As described previously [25], total RNA was isolated at 1, 3, 7 and 14 days after MCAO using Trizol reagent (Invitrogen, USA) and then reversed transcribed to cDNA using a PrimeScript RT Reagent Kit (Takara, Dalian, China). Quantitative PCR was performed by an ABI StepOnePlus Real-time PCR System (Applied Biosystems, USA) with fluorescent dye (SYBR Green I, Takara). Protocol of the real-time PCR was conducted as follows: holding stage at 95 °C for 30 s, followed by 95 °C for 5 s, 60 °C for 34 s, 40 cycles. To obtain a melting curve as follows: holding at 95 °C for 15 s, cooling to 60 °C for 1 min, and then heating slowly at 0.3 °C/s until 95 °C for 15 s. The primer sequences were as follows: VEGF: F: CGA CAG AAG GGG AGC AGA AAG and R: GCA CTC CAG GGC TTC ATC ATT; Ang-1: F: GTC ACT GCA CAA AAG GGA CA and R: GGC TTA CAA GGA TGG CGT TA; VEGFR-2: F: TAGCACGACAGAGACTGTGAGG and R: TGAGGTGAGAGAGATGGGTAGG; Tie-2: F: TGCCACCATCACTCAATACC and R: AAACGCCAATAGCACGGTGA; CD31: F: TTTCGCTGCCAAGCTGGCGT and R: CCACCTGCACGCTGCACTTGAT; α-SMA: F: TGGCCACTGCTGCTTCCTCTTCTT and R: GGGGCCAGCTTCGTCATACTCCT; and GAPDH: F: CTC TAA GGC TGT GGG CAA GGT CAT and R: GAG ATC CAC CAC CCT GTT GCT GTA. For GAPDH constantly express in the brain, so we select it as comparator in our experiment. The relative mRNA expression were normalized to GAPDH levels and analyzed using the 2^−ΔΔCT^ method.

### Western blotting

The rats were euthanized and decapitated, and their ischemic ipsilateral cortices were rapidly dissected on ice and stored at −80 °C until needed. The samples were then taken from the freezer and sonicated in RIPA buffer (Beyotime Institute of Biotechnology, China) containing phosphatase and protease inhibitor cocktails. A BCA Kit (Beyotime Biotechnology) was then used to determine the protein concentrations. Equal amounts of sample were separated by SDS-PAGE and transferred onto PVDF membranes, which were blocked in 5% non-fat dry milk at room temperature for 1 h. The membranes were then incubated overnight at 4 °C with primary antibodies against VEGF (1:1000 Bioworld Technology, USA), Ang-1 (1:1000 Bioworld Technology, USA), Shh (1:250 Life Technology, USA), Patched-1 (1:1000 Abcam, UK), Smoothened (1:1000 Abcam, UK) and Beta-actin (1:5000 Bioworld, USA). The next day, the membranes were incubated with the appropriate HRP-conjugated secondary antibodies for 2 h at room temperature. Immunoreactive bands were visualized with chemiluminescence reagents that were provided with an ECL kit (Bioworld, USA). Blot intensity was detected using Image J software.

### Immunofluorescence staining

The animals were sacrificed via cardiac perfusion with 0.9% saline, and their brains were fixed in 4% paraformaldehyde before being cut into 20 μm coronal sections. Double-immunofluorescence staining was employed in order to specifically for identifying endothelial cells (EC) and pericytes colocalized vessels in ischemia boundary zones (IBZs). CD31 is an endothelial cell marker, and α-smooth muscle actin (α-SMA) is a pericyte marker. For CD31 and α-SMA-positive vessel density quantification, the brain slices were incubated with anti-CD31 (1:200, Abcam, USA) and anti-α-SMA (1:50, Santa Cruz, CA, USA) antibodies overnight at 4 °C. After being washed with phosphate buffer solution (PBS), the sections were incubated with the appropriate secondary antibodies for 2 h in the dark at room temperature. Cell nuclei were stained with DAPI. Five slides of each brain were selected, and each slide contained three to four fields of view of the IBZ (ischemic boundary zone). To analyze EC proliferation, we calculated the percentage of Brdu-positive endothelial cells in the IBZ. The brain sections were imaged using an Olympus microscope with a BX51 digital camera (Olympus, Japan), and the images were quantified with IPP 6.0 software. All evaluations were performed by a blinded investigator.

### Statistical analysis

All data are presented as the mean ± standard error (SE) and were analyzed using SPSS 16.0 statistical analysis software (SPSS, Chicago, IL, USA). Student’s t-test was used to assess the differences between two groups. Differences among multiple groups were analyzed via one-way analysis of variance (ANOVA). *P* < 0.05 was considered statistically significant. All evaluations were performed in a blinded manner.

## Results

### PTS attenuates ischemic brain injury in rats

**Fig. 1:**
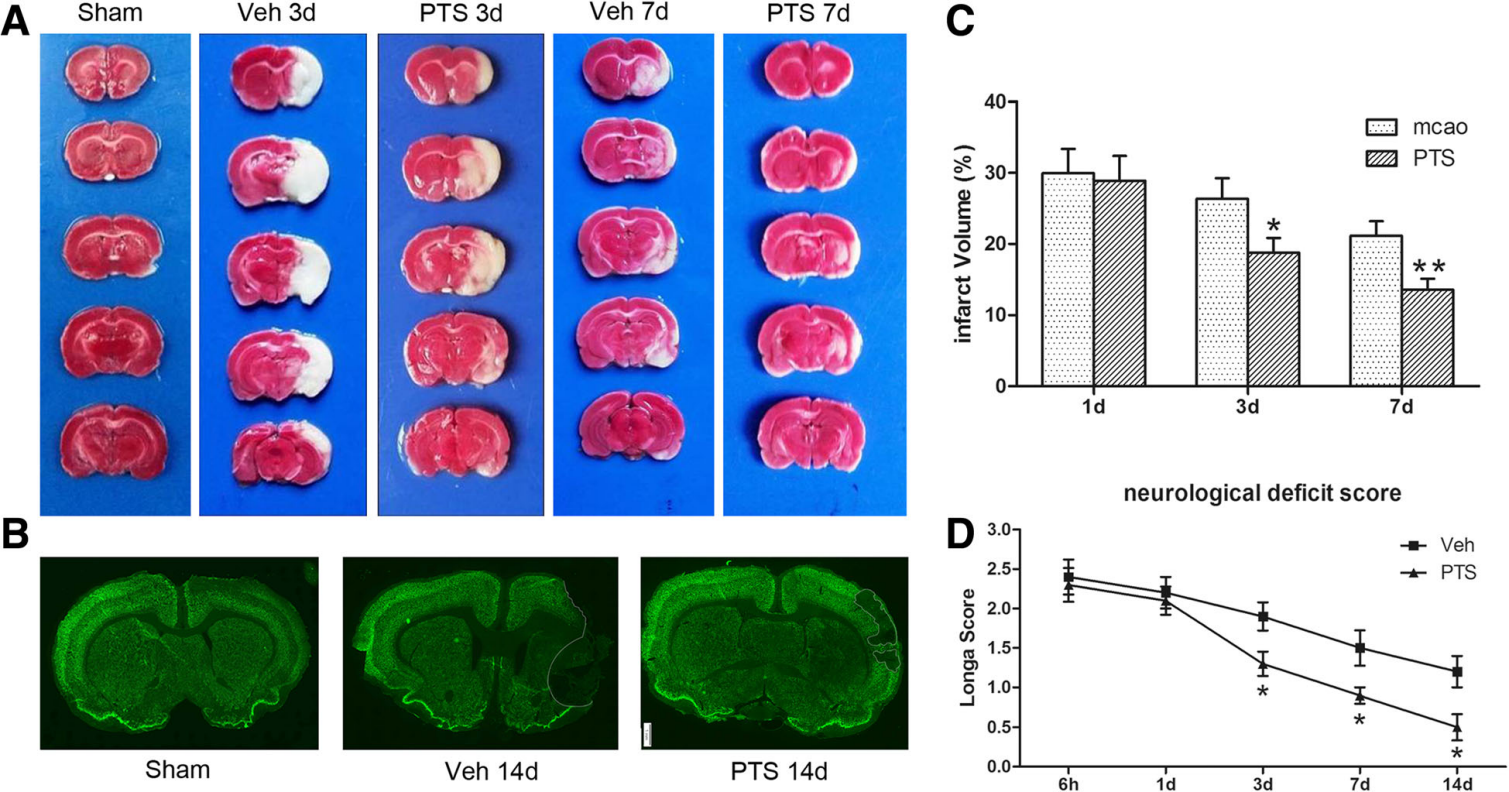
PTS protects against ischemia-induced brain injury. PTS can reduce brain infarct volumes and improve neurological deficit scores after MCAO in rats. **a** TTC staining showing infarct volumes at 3 and 7 days after surgery. Normal brain tissue appears red, and infarcted tissue appears pale gray. **b** Neuron immunofluorescence staining (*green*) showing neuronal loss at 14 days after surgery, **c** Quantitative analysis of brain infarct volumes in vehicle- and PTS-treated rats at 1, 3, 7 days after MCAO. *P < 0.05, **P < 0.01 versus Veh group, *n* = 6 per group. **d** Neurological deficit scores in the two groups were assessed by the Longa scale scoring system at 6 h, 1, 3, 7, and 14 days after reperfusion.*P < 0.05 versus Veh group, *n* = 10 per group

To explore the effects of PTS on ischemic brain injury, we assessed infarct volumes and neurological deficit scores. TTC staining indicated that PTS administration significantly reduced infarct volumes in treated rats compared to vehicle rats at 3 and 7 days after surgery (Fig. 1a and c). Immunofluorescence staining showed that neuron loss was significantly decreased in PTS rats at 14 days after cerebral ischemia-reperfusion (Fig. 1b). Neurological deficit score results showed that PTS can improve neurological function at 3 days after surgery and that this effect lasts for 14 days (Fig. 1d). These results confirmed that PTS exerts neuroprotective effects in the setting of experimental ischemic stroke-induced brain injury.

### PTS enhances cerebral perfusion in ischemic brain tissue

**Fig. 2:**
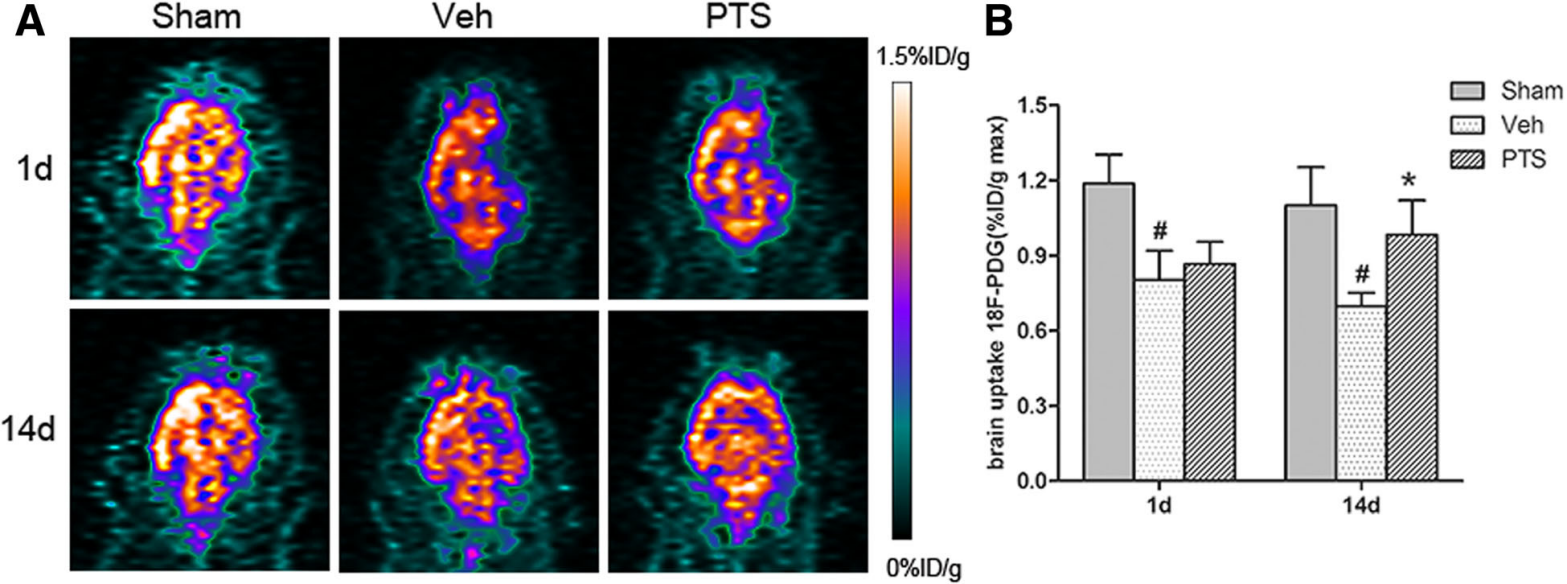
PTS enhances cerebral perfusion in stroke rats. **a** ^18^F-FDG micro-PET image showing brain perfusion in the different groups at 1 and 14 days after MCAO. **b** Quantitative analysis of ^18^F-FDG uptake in the different groups at 1 and 14 days after cerebral reperfusion. Data are presented as the mean ± SEM, #*P* < 0.05 versus Sham group; *P < 0.05 versus Veh group, *n* = 6 per group

PET with ^18^F-FDG is a powerful and noninvasive tool for assessing the effects of cerebrovascular disease therapy [26]. Micro-PET/CT was performed to determine whether PTS can improve cerebral blood flow in rats after MCAO. The results showed that ^18^F-PDG uptake was significantly decreased in ischemic brain tissue at 1 day after MCAO (Fig. 2a and b). Interestingly, at 14 days after surgery, ^18^F-PDG uptake was significantly higher in the PTS group than in the vehicle group (Fig. 2a and b). These results show that PTS can enhance cerebral perfusion in the ischemic boundary zones of experimental stroke rats.

### PTS promotes angiogenesis factor expression in ischemic cortical tissue

**Fig. 3:**
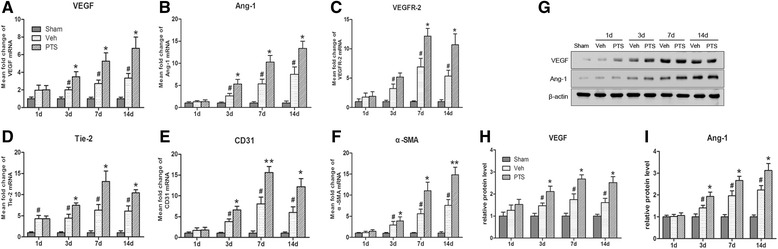
PTS promotes angiogenesis factor expression in ischemic lateral cortical tissue. PTS promotes angiogenesis factor expression in ischemic lateral cortical tissue at different time points. The mRNA levels of VEGF (**a**), Ang-1 (**b**), VEGFR2 (**c**), Tie-2 (**d**), CD31 (**e**), and α-SMA (**f**) were determined by real-time PCR at 1, 3, 7, and 14 days after MCAO. Data are presented as the mean ± SEM, #*P* < 0.05 versus Sham group; *P < 0.05, **P < 0.01 versus Veh group. *n* = 6 per group. **g** The protein levels of VEGF and Ang-1 were measured by western blotting. (**h**-**i**) Quantitative analysis of VEGF and Ang-1 expression. #*P* < 0.05 versus Sham group; *P < 0.05 versus Veh group. *n* = 6 per group

The relative levels of VEGF, Ang-1, VEGFR-2, Tie-2, CD31 and α-SMA mRNA expression in ischemic lateral cortical tissue were measured using real-time PCR. The results showed that VEGF and Ang-1 expression was increased after MCAO and that PTS can significantly increase VEGF and Ang-1 mRNA expression at 3, 7 and 14 days after surgery in treated rats compared to vehicle rats (Fig. 3a and b). These results were consistent with those of a prior report [27]. VEGFR-2 and Tie-2 expression was also increased at different time points after surgery (Fig. 3c and d). The mRNA expression levels both receptors peaked at 7 days before decreasing at 14 days after surgery. PTS administration significantly increased their expression at 3, 7 and 14 days after surgery. PTS also up-regulated the levels of CD31 and α-SMA mRNA expression in treated rats compared to vehicle rats beginning at 3 days after surgery. These increases persisted until 14 days after cerebral ischemia-reperfusion (Fig. 3e and f). In addition, the levels of VEGF and Ang-1 protein expression were also detected by western blotting. VEGF expression tended to increase after MCAO, peaking at 7 days and then decreasing at 14 days after surgery. These results were consistent with those of a previous study [23], Ang-1 protein expression tended to increase after MCAO, and PTS significantly increased both VEGF and Ang-1 protein expression in ischemic lateral cortical tissue in treated rats compared with vehicle rats at 3, 7 and 14 days after surgery (Fig. 3g, h, i).

### PTS increases angiogenesis in ischemic cortical tissue

**Fig. 4:**
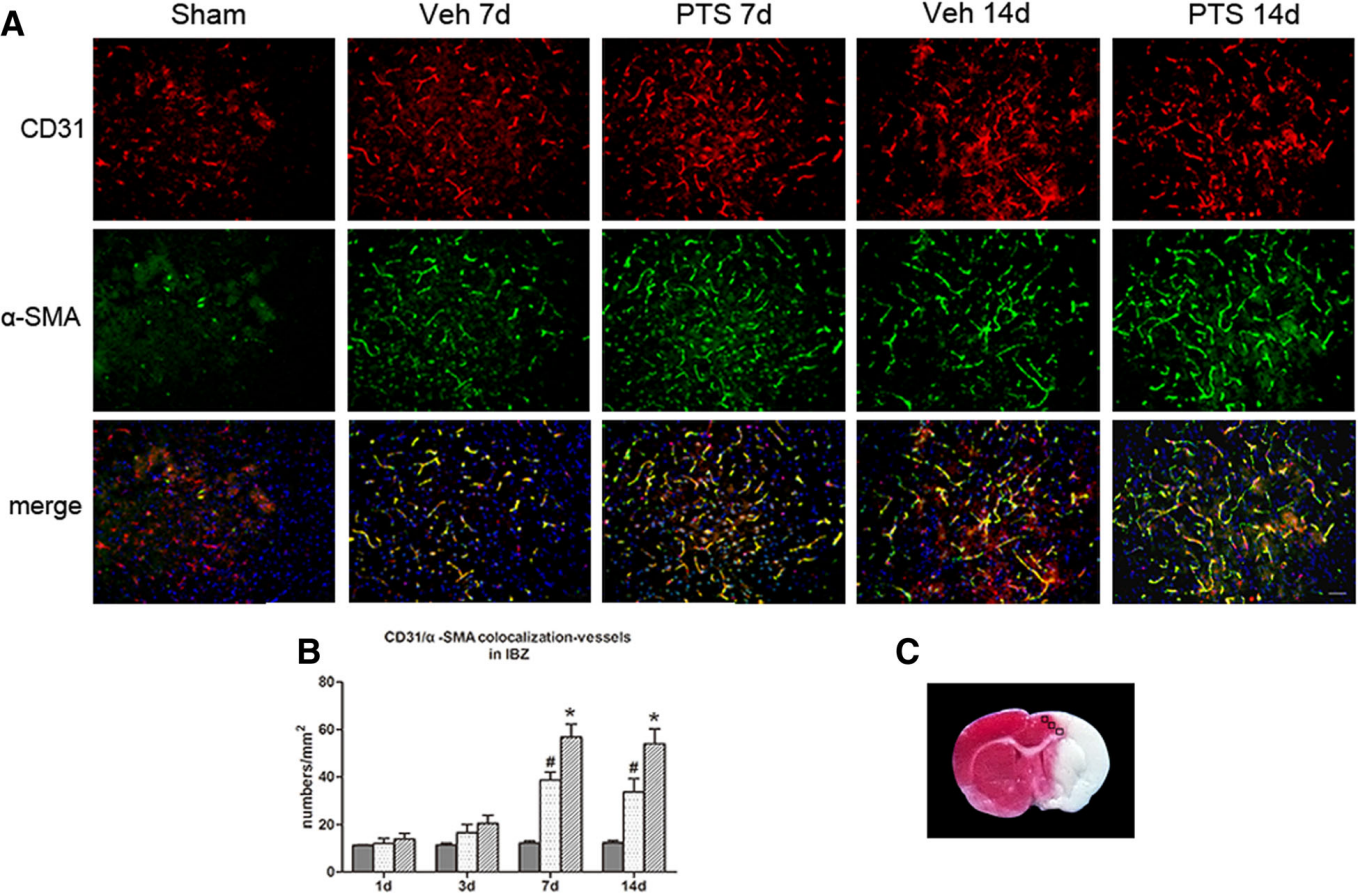
PTS increases microvascular density in ischemic cortical tissue. PTS can increase CD31 and α-SMA colocalization in ischemia boundary zones. **a** CD31 (*red*) and α-SMA (*green*) double-immunostaining in the ischemia boundary zone (IBZ) at 7 and 14 days after surgery. Scale bar = 50 μm. **b** Quantitative analysis of the microvessels in the IBZ. Data are presented as the mean ± SEM. #*P* < 0.05 versus Sham group; *P < 0.05 versus Veh group. *n* = 6 per group. **c** The typical detected areas of CD31 and α-SMA colocalization in boxed zones of brain slices

Accumulating evidence indicates that angiogenesis plays an important role in ischemic stroke-induced brain injury repair and long-term functional recovery [3] A previous study reported that capillary sprouting and vessel growth continue for at least 3 weeks after ischemic injury in ischemic boundary zones [28]. In this study, to evaluate the effect of PTS on ischemic brain microcirculation, we examined the expression of the endothelial cell marker CD31 and the pericyte marker α-SMA in ischemic ipsilateral cortical tissue via immunostaining at different time points. The results showed that ischemia substantially induces angiogenesis in brain penumbra in rats and that vessel density peaks at 7 days before decreasing at 14 days after surgery. CD31 and α-SMA colocalizationed vessels density was significantly higher in the PTS group than in the vehicle group at 7 and 14 days after surgery (Fig. 4a-b). It is known that the processes of post-stroke neurogenesis and angiogenesis are linked together [29]; therefore, PTS may improve cerebral perfusion and promote neural functional recovery by promoting angiogenesis.

### PTS promotes endothelial cell proliferation after MCAO

**Fig. 5:**
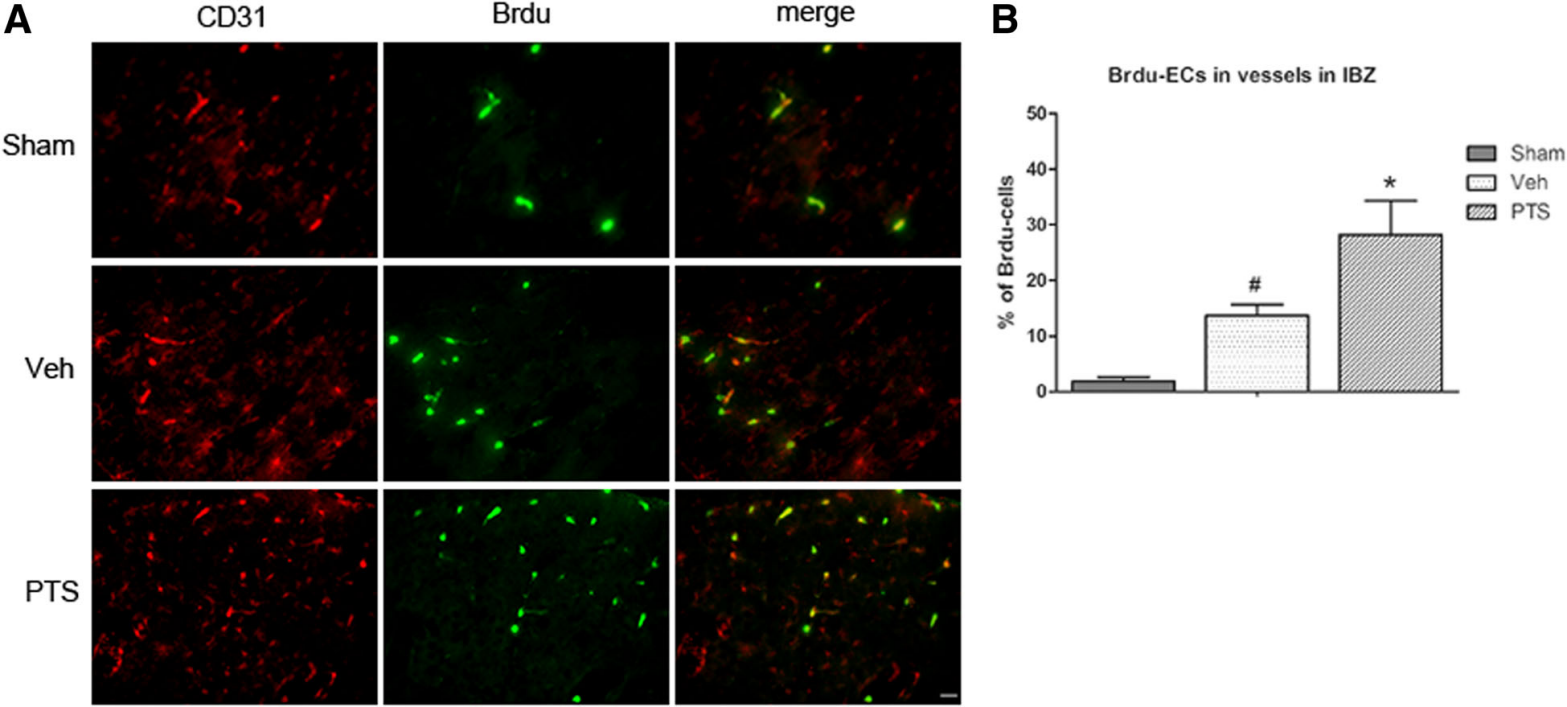
PTS enhances vascular endothelial cell proliferation in ischemic cortical tissue. PTS can increase the number of CD31- and Brdu-double-immunostained cells in ischemic lateral cortical tissue after MCAO. **a** CD31 (*red*) and Brdu (*green*) double-immunostaining in the ischemia boundary zone (IBZ) at 14 days after surgery. Scale bar = 20 μm **b** Quantitative analysis of colocalized endothelial cells in the IBZ. #*P* < 0.05 versus Sham group; *P < 0.05 versus Veh group. *n* = 6 per group

To verify the effects of PTS on endothelial cell proliferation after MCAO, we intraperitoneally injected rats with Brdu, a thymidine analog that is incorporated into the DNA of dividing cells during S-phase. CD31 and Brdu double-staining was used to mark newborn endothelial cells, as shown in Fig. 5a and b. EC proliferation was observed in the IBZ after surgery, and PTS apparently increased the numbers of CD31- and Brdu-double-stained cells in the treated group compared with the vehicle group at 14 days after surgery, confirming that PTS promotes EC proliferation after ischemic stroke.

### PTS modulates pro-angiogenic factor expression by activating the Shh pathway

**Fig. 6:**
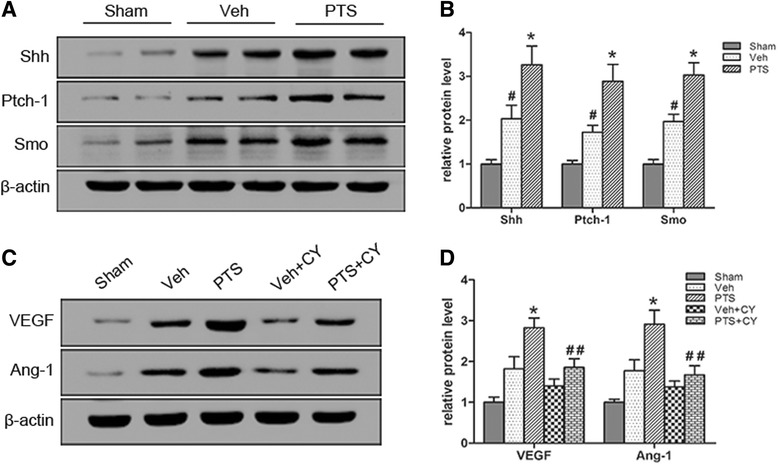
PTS modulates VEGF and Ang-1 expression through Shh pathway activation. **a** Shh pathway component levels were measured by western blotting at 7 days after surgery. **b** Quantitative analysis of Shh, Ptch-1 and Smo expression. #*P* < 0.05 versus Sham group; *P < 0.05 versus Veh group. *n* = 6 per group. **c** The protein levels of VEGF and Ang-1 were measured by western blotting in the different groups at 7 days after surgery. **d** Quantitative analysis of VEGF and Ang-1 expression. *P < 0.05 versus Veh group. ##*P* < 0.01 versus PTS group; *n* = 6 per group

To elucidate the mechanism underlying PTS-induced increases in angiogenesis, Shh pathway activity was detected by western blotting. The expression of Shh and two transmembrane receptors, Ptch-1 (Patched-1), Smo (Smoothened), was detected in the study. As shown in Fig. 6a and b, PTS up-regulated Shh activity and Ptch-1 and Smo expression in the treated group compared with the Sham group at 7 days after cerebral reperfusion. To determine whether PTS increases VEGF and Ang-1 expression via the Shh pathway, PTS was administered with and without the specific Shh pathway inhibitor CY (cyclopamine). As shown in Fig. 6c and d, the levels of VEGF and Ang-1 protein expression were dramatically decreased in cyclopamine-treated rats. These data indicate that PTS at least partially regulates VEGF and Ang-1 expression via the Shh pathway.

## Discussion

In the current study, we demonstrated that PTS administration can reduce brain infarction volumes and improve neurological deficits in a rat focal ischemic injury model. Furthermore, we showed that PTS can improve cerebral blood perfusion and that the mechanism underlying these effects may be associated with Shh pathway activation and VEGF and Ang-1 expression regulation.

Ischemic stroke, which is induced by cerebral artery occlusion, dramatically decreases local cerebral blood flow, causing cell death, brain atrophy, and functional deficits. Restoration of local blood perfusion in ischemic brain tissue plays a key role in tissue repair and functional recovery [30]. The early response facilitates cerebral blood flow recovery through collateral circulation and anastomotic vessel activation, and the long-term response involves angiogenesis [31]. Angiogenesis is defined as the growth of new vessels from pre-existing blood vessels, which occurs in the ischemic boundary zones of brain infarct regions. Mounting evidence indicates that angiogenesis is involved in neurological functional recovery and can improve cerebral microcirculation perfusion and reduce infarct volumes [23, 29]. In our study, PTS administration significantly improved rat neurological deficit scores, reduced infarct volumes at 3, 7 and 14 days after MCAO, enhanced ^18^F-PDG uptake in ischemic brain tissue, and increased cerebral perfusion after surgery. These results confirmed that PTS exerts protective effects on ischemic brain injury.

Angiogenesis is a multi-step biological process involving the proliferation and sprouting of endothelial cells, the formation of tube-like vascular structures, and the branching and anastomosis of new vessels. Many cytokines take part in this complicated process, such as VEGF, Ang-1, HIF-1, bFGF, and EPO. VEGF and Ang-1 play the most prominent roles in angiogenesis after ischemic injury. VEGF is probably the most important angiogenesis inducer during the early stages of recovery from ischemic stroke. VEGF promotes angiogenesis by up-regulating VEGFR-2 expression [32]. VEGF binds to VEGFR-2 and then triggers EC mitotic and migratory processes to facilitate tube formation [33]; however, this process may increase microvessel leakage and induce brain edema. Ang-1 promotes angiogenesis and new vessel remodeling, and also acts as an anti-permeability factor, Ang-1 binds to its specific receptor Tie-2 and recruits mural cells to wrap around endothelial cells, thereby ensuring the eventual maturation and stabilization of new blood vessels [34]. Therefore, VEGF and Ang-1 exert synergistic effects on angiogenesis after stroke [35]. A previous study showed that ECs begin mitosis as early as 1 day after mouse cerebral reperfusion after 30 min of MCAO and that the number of vessels begins to significantly increase on the third day before peaking at 7 days after stroke. The levels of VEGF and Ang-1 were increased, too [28]. In addition, hydrogen sulfide increases VEGF and Ang-1 expression in ischemic brain tissue at 7 days after MCAO, as well as the numbers of Brdu- and CD31-double-positive cells in the ipsilateral hemisphere [1]. In this study, CD31 and α-SMA colocalization was significantly higher in the PTS group than in the vehicle group at 7 and 14 days after MCAO, confirming that PTS significantly promotes angiogenesis and enhances neovascular integrity. We also examined VEGF/VEGFR2 and Ang-1/Tie-2 expression in ischemic brain cortical tissue. The results showed that VEGF and VEGFR2 mRNA levels were increased after PTS injection at different time points, findings that were consistent with those regarding Ang-1 and Tie-2 expression. In addition, western blotting confirmed that the levels of VEGF and Ang-1 protein expression were also significantly increased in the PTS group at different time points after surgery.

The study confirmed that PTS can effectively promote angiogenesis after ischemic stroke in rats, but the mechanism underlying the effects of PTS is unknown. Previous studies have reported that VEGF can be regulated by multiple signaling pathways, such as the PI3K/AKT [36], Apelin-APJ [37], and DLL4/Notch signaling pathways [38]. However, few studies have examined the mechanism underlying upstream Ang-1 regulation. A recent study showed that the Shh signaling pathway can co-regulate two angiogenic cytokine families, namely, those to which VEGF and Ang-1 and Ang-2 belong [15]. The Shh signaling pathway is an evolutionarily conserved developmental signaling cascade that regulates multiple biologic processes, such as embryonic development, injury repair, and angiogenesis [39]. Shh transmits its signal by binding to the plasma membrane receptor Ptch-1, leading to Ptch-1 internalization and degradation, Smo de-repression, and downstream transcription factor activation [40]. In addition, Shh exerts protective effects in several animal ischemia/reperfusion models, including cerebral and myocardial infarction and hind-limb ischemia models [41, 42]. A previous study showed that endogenous Shh expression is up-regulated in hippocampal neurons after focal ischemia and that cyclopamine, an Shh pathway inhibitor, can suppress post-ischemic sub-granular neural progenitor cell proliferation [43]. Shh can increase VEGF, Ang-1, and Ang-2 expression in astrocytes after oxygen–glucose deprivation (OGD). Moreover, a previous study showed that inhibiting the Shh signaling pathway with 5EI decreased VEGF, Ang-1, and Ang-2 expression [44]. The current study illustrated that PTS up-regulated Shh, Ptch-1 and Smo expression after reperfusion and also increased the levels of VEGF and Ang-1 protein expression. In addition, the Shh pathway could be inhibited by cyclopamine, and cyclopamine-treated rats exhibited dramatically decreased VEGF and Ang-1 protein expression. These data demonstrate that PTS activates the Shh pathway after ischemia-reperfusion injury, which may contribute to its neuroprotective effects.

## Conclusion

In summary, our study confirmed that PTS enhances cerebral blood flow, promotes cerebral angiogenesis and modulates VEGF and Ang-1 expression. The molecular mechanism underlying the effects of PTS may be associated with Shh pathway activation. PTS may be a promising drug for ischemic stroke treatment.

## Additional files

Additional file 1: Figure S1. HPLC chromatogram of PTS. The ginsenoside Rg1, notoginsenoside R1, and ginsenoside Re are the main components of PTS with the the concentrations of ≥ 50%, ≥ 11% and ≥ 6% respectively. (DOC 50 kb)
Additional file 2: Figure S2. In a previous study, different doses of PTS (25, 50 and 100 mg/kg/day) were used after MCAO rats. (**a**) Quantitative analysis of brain infarct volumes in four groups at 3 d after MCAO. *P<0.05, versus Veh group, n = 10 per group. (**b**) Neurological deficit scores in the four groups were assessed by the Longa scale scoring system at 6 h, 1 d, 3 d after reperfusion.*P<0.05 versus Veh group, n = 10 per group. (TIF 318 kb)

## Abbreviations

Brdu: Bromodeoxyuridine
CY: Cyclopamine
IBZ: Ischemic boundary zone
MCAO: Transient middle cerebral artery occlusion
PET: Micro-positron emission tomography
Ptch-1: Patched-1
PTS: Panaxatriol saponins
Shh: Sonic hedgehog
Smo: Smoothened
TTC: Triphenyltetrazolium chloride
α-SMA: α-smooth muscle actin
