# Supplementary figures and images for: Panaxatriol saponins promotes angiogenesis and enhances cerebral perfusion after ischemic stroke in rats
Source: BMC Complement Altern Med. 2017 Jan 23;17:70. doi: 10.1186/s12906-017-1579-5 (PMC5259846; doi:10.1186/s12906-017-1579-5)

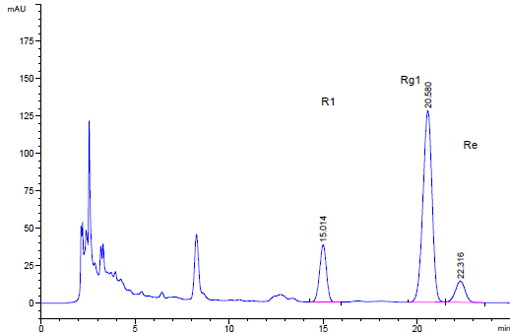

Supplement: Additional file 1: Figure S1. — HPLC chromatogram of PTS. The ginsenoside Rg1, notoginsenoside R1, and ginsenoside Re are the main components of PTS with the the concentrations of ≥ 50%, ≥ 11% and ≥ 6% respectively. (DOC 50 kb) [file 12906_2017_1579_MOESM1_ESM.tif]

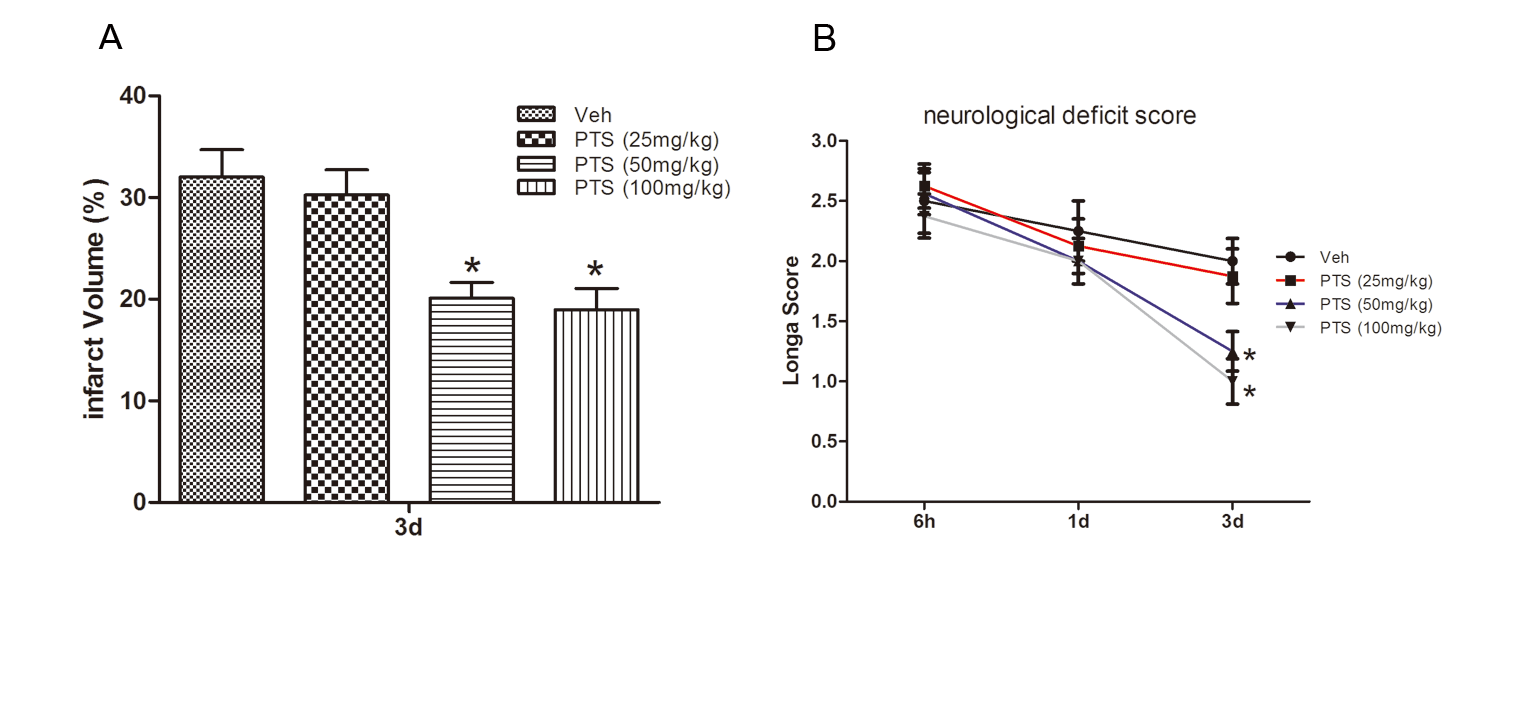

Supplement: Additional file 2: Figure S2. — In a previous study, different doses of PTS (25, 50 and 100 mg/kg/day) were used after MCAO rats. (a) Quantitative analysis of brain infarct volumes in four groups at 3 d after MCAO. *P<0.05, versus Veh group, n = 10 per group. (b) Neurological deficit scores in the four groups were assessed by the Longa scale scoring system at 6 h, 1 d, 3 d after reperfusion.*P<0.05 versus Veh group, n = 10 per group. (TIF 318 kb) [file 12906_2017_1579_MOESM2_ESM.tif]
